# Sex-specific analysis of hiking accidents in the Austrian Alps: a follow-up from 2015 to 2021

**DOI:** 10.3934/publichealth.2024008

**Published:** 2024-01-31

**Authors:** Linda Rausch, Mirjam Limmer, Elena Pocecco, Gerhard Ruedl, Markus Posch, Martin Faulhaber

**Affiliations:** 1 Department of Sport Science, University Innsbruck, 6020 Innsbruck, Tyrol, Austria; 2 Institute of Outdoor Sports and Environmental Science, German Sports University Cologne, 50933 Cologne, North Rhine-Westphalia, Germany; 3 Austrian Society of Alpine and High-Altitude Medicine, 6414 Mieming, Tyrol, Austria

**Keywords:** mountain hiking, sports epidemiology, mountain accidents, sex differences, injury prevention, alpine sport

## Abstract

**Background:**

Hiking is one of the most popular leisure sport activities practiced in the Alps during the summer season, but bears the risk of mountain emergencies, accidents, and fatalities. This paper provides an updated analysis of hiking accidents for the years 2015 to 2021 in the Austrian Alps, thereby outlining fatal and non-fatal accident characteristics.

**Methods:**

For this retrospective analysis, mountain hiking accidents documented by the Austrian Alpine Police during a 7-year period were screened for potential exclusion criteria. The final sample size consisted of 7368 accidents and 7552 victims. The outcome measures were mainly specified by sex, age, injury degree, injury location, pathophysiological characteristics, and cause of injury.

**Results:**

The overall annual number of accidents showed a continuous increase from 428 in 2015 to 544 in 2021. In total, 7.1% of the total victims died during the 7-year period, with male hikers being significantly more affected than female hikers (m: 80.8%, f: 19.2%; p ≤ 0.001). The sex specific distribution for non-fatal hiking accidents was 55.9% in women and 44.1% in men. Male victims showed significantly more frequent cardiovascular events (m: 78.5%, f: 21.5%), multiple injuries (m: 60.2%, f: 39.8%), and wounds/blood loss (m: 57.4%, f: 42.6%) than female victims, whereas women showed more fractures (m: 31.5%, f: 68.5%) than men (p ≤ 0.001). Additionally, men were more likely to injure their abdomen/chest (3.7%), head (14.1%), and multiple body parts (26.5%), whereas women were more likely to injure their ankle or foot (42.3%). Finally, men were more likely to have an accident during the ascent (24.1%), whereas women during the descent (69.0%) (p ≤ 0.001).

**Conclusion:**

This paper provides the latest data and a deeper insight into sex-specific characteristics of mountain hiking accidents in the Austrian Alps.

## 1. Introduction

The frequency of visits to mountainous areas across every continent has been significantly increasing in the past decade, with hiking being the most popular mountain sport activity worldwide [1]. The most visited mountain areas include the Alps, which is the highest and most extensive mountain range system located in south-central Europe, spanning across eight countries, with an annual number of 120 million people visiting the most popular regions in Switzerland, France, Italy, and Austria [2]. In Austria, millions of people hike annually on marked trails and paths in the Alps during the summer season [3]. According to the Tyrolean Declaration on Best Practice in Mountain Sports, hiking includes all forms of walking on trails and footpaths in the alpine countryside, excluding passing glaciers or rock climbing [4]. Nevertheless, hiking bears the risk of mountain emergencies, accidents, and fatalities [5]. Recent studies have investigated the circumstances and causes of fatalities in hikers [5], as well as the characteristics of non-fatal hiking accidents in the Alps [6]. The majority of fatal and non-fatal hiking accidents occurred because of falls mainly during descent [5],[6].

The most common causes of fatal hiking accidents outcomes included neck fracture, chest trauma, blood loss, and traumatic brain injuries [5]. The level of altitude was only associated with traumatic brain injuries, which occurred more often above 1800 m and were related to the height of the fall [5]. Reported fatalities were accompanied by equipment shortages, especially below 1800 m [5]. The lack of adequate boots, clothing, and a backpack was negatively associated with the years of hiking experience, regardless of the altitude level [5]. Fatal accidents seem to be independent of age and occurred more frequently in a pathless terrain [7].

It has been consistently shown that the prevalence of fatal hiking accidents differed between sexes [5],[7],[8]. Between 2003 and 2018, 87% of fatal accidents were reported in male hikers and 22% in female hikers in the Swiss Alps [5]. Similar sex distributions were reported between 2003 and 2014 in the Austrian Alps [7] and between 1970 and 2014 in the Canadian mountainous terrain [8].

According to two studies between the years 2003 and 2018, the prevalence of non-fatal fall-related accidents in the Austrian Alps was reported to be similar between the sexes, with approximately 50–60% in women and 40–50% in men [6],[7]. The most common injury locations involved were the ankle joint, the head, and the lower leg [6]. Women were more likely to injure the ankle than men, whereas men seemed to suffer more often from multiple injuries compared to women [6]. Impaired vision was defined as a dominant characteristic in fall-related non-fatal accidents for both sexes [7].

Literature on hiking accidents mostly investigated fall-related accidents and focused either on fatal or on non-fatal outcomes in its sex-specific analysis [5]–[7]. Faulhaber et al. [6] reported significant sex-differences concerning circumstances of falls from hikers who experienced fatal and non-fatal accidents in the Austrian Alps. However, to the best of our knowledge, a report including all hiking accidents within the past few years outlining sex-specific analyses of accident causes and types has been lacking. According to Van Mechelen et al. [9], the establishment of the etiology and mechanisms of injuries is the prerequisite for the introduction of evidence-based preventive measures; the assessment of sex-specific characteristics related to hiking accidents seems to be essential for the development of effective recommendations, specifically for female and male hikers. Thus, the aim of this study is to provide an updated analysis of hiking accidents between the years 2015 and 2021 in the Austrian Alps, thereby outlining fatal and non-fatal accident characteristics including hiking circumstances, locations, types, injury degrees, and causes, while focusing on potential sex differences. The details of this sex-specific analysis could provide the basis for strategy development in order to improve the safety of hikers, as well as risk management and prevention of future accidents.

## 2. Materials and methods

### 2.1. Study design and data source

This study was designed as a retrospective analysis of documented mountain hiking accidents that occurred during the summer seasons (May 1st – October 31st) within a 7-year period (2015–2021) in the Austrian Alps. According to earlier studies [6],[7], the data was retrieved from the database for mountain accidents and emergencies, which is administered by the Austrian Alpine Police as part of the Austrian Ministry of the Interior. After gaining approval by the Austrian Alpine Police authority, members of the Austrian Board of Alpine Safety accessed the database and provided an anonymized dataset for analyses. Then, these analyses were carried out by staff members of the Department of Sport Science (University of Innsbruck, Austria). The dataset included all accidents (non-fatal and fatal) which were documented after receiving an emergency call either via the local mountain rescue service or via an emergency call center. The staff of the Austrian Alpine Police continuously documented details of emergencies and accidents during their routine work using standardized software forms. The details of the reports included the date, time and location of the accident, the type of activity which led to the accident (e.g., hiking, climbing ...), a short description of the accidents' circumstances, and the number of victims involved.

For each victim, the following characteristics were reported: sex, age classified in categories (≤15, 16–34, 35–54, 55–74 and ≥75) for data analysis, alcohol intake on the day of the accident (yes, no, not specified), rescue by helicopter and/or terrestrial rescue, type of trail (drive ways or forest roads, marked hiking trail or path, pathless terrain, other conditions/not specified), and accident happening during the ascent or descent. Furthermore, the report specified the injury cause (falling/tripping/stumbling, exhaustion, cardiac event/circulatory disturbances, getting lost, falling rocks, unspecified disease, other), injury degree (unharmed, slightly injured, seriously/life-threatening injured, dead, not specified), injury type (strain/contusion, dislocation, fracture, open wound/bleeding, exhaustion, cardiac event/circulatory disturbances, not specified), and injury location (polytrauma/multiple injury locations, head, shoulder/back, chest/abdomen, arm/hand, hip/pelvis, thigh, knee, lower leg, ankle/foot), as described in a previous study [7]. Before the analyses, the dataset was comprised of a total of 8825 accidents and the characteristics of 9847 hikers involved.

The study was performed in conformity with the ethical standards of the 2013 version of the Declaration of Helsinki and was approved by the Institutional Board for Ethical Questions in Science of the University of Innsbruck, Austria (Certificate No 21/2021).

### 2.2. Data screening and data selection

An initial dataset screening was performed by all six authors to identify potential accidents which occurred during any other activity than hiking. Potentially excluded accidents were discussed, and the exclusion criteria were finalized by three authors (ML, LR, MF) and then reapplied to the whole dataset by two authors (ML, LR). Based on Faulhaber et al. (2017) [7], the exclusion criteria were defined as follows: (1) mountain activities other than mountain hiking (i.e., high-altitude mountaineering, climbing/fixed-rope climbing, and trail running/mountain running), (2) further activities (i.e., mushroom/berry collecting, taking photographs, accidents in/around a mountain hut, playing children...), (3) professional activities (i.e., hunting, mountain guiding, alpine farming...), and (4) other activities (i.e., mountain rescue without victims/persons involved, human remains without information on the accident, double or miss-entry of the accident).

**Figure 1.**
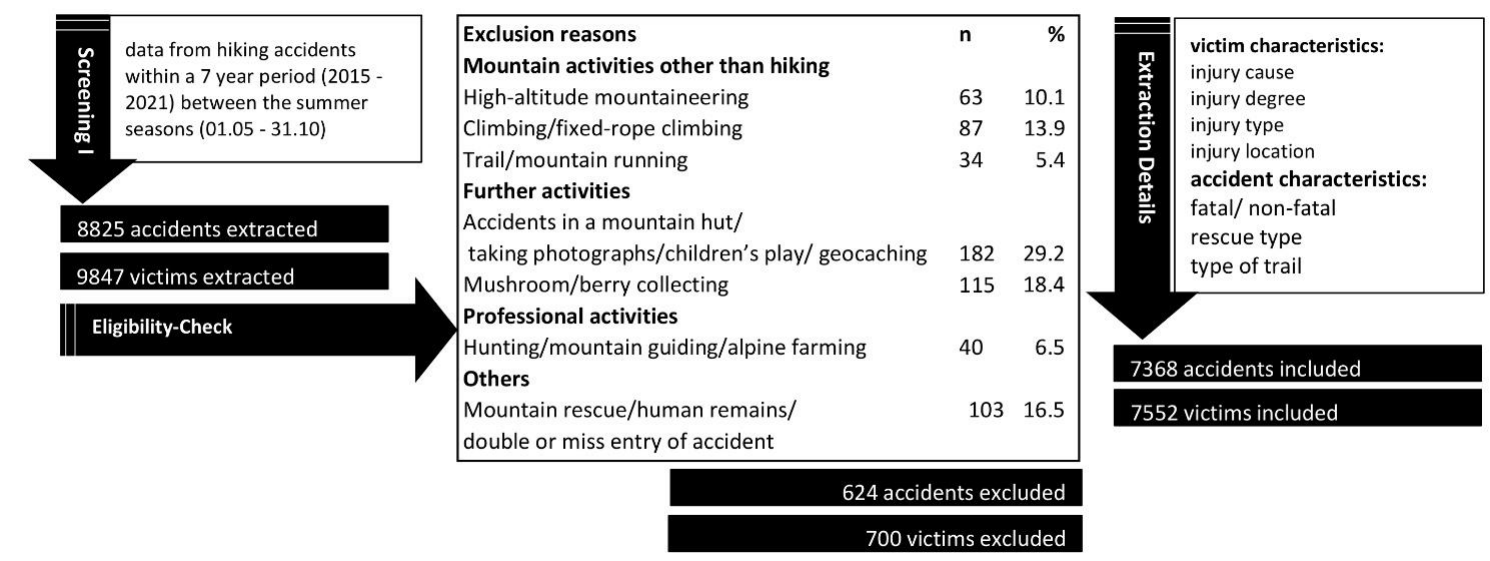
Process of data screening, selection and extraction.

By applying the exclusion criteria, a second dataset screening was performed by two authors (ML, LR). In the case of a disagreement between the two authors, a third person (MF) was consulted. In Figure 1, the process of data screening, selection, and extraction is summarized. After applying the exclusion criteria, 624 accidents and the characteristics of 700 victims were excluded from the consecutive analyses (Figure 1).

### 2.3. Statistics

In order to run the statistical analyses, the SPSS software (V.26, IBM) and Microsoft Excel (Microsoft Office Professional Plus, 2019) were used. Chi-Square Tests were conducted to test for independence of sex and all accident-related variables such as the cause, fatality, condition, and terrain of the accident, as well as the injury degree, type, and location resulting from the accident. When comparing more than 2 categories, Cramer's V was used to estimate the effect size. For the comparison of 2 categories, the Phi coefficient (Φ) was used. Considering the effect size for post-hoc analyses following the Chi-Square Test, the Bonferroni adjustment was applied on adjusted residuals to determine where the largest sex differences between observed and expected outcomes appeared. The effect sizes were documented according to Cohen et al., with either small (V/Φ = 0.1), medium (V/Φ = 0.3) or large (V/Φ = 0.5) effect sizes. The results were presented as absolute and relative values for each victims' variable. P values < 0.05 were considered to indicate statistical significance.

## 3. Results

### 3.1. Annual number of victims characterized by age, sex, and fatality outcome

**Figure 2.**
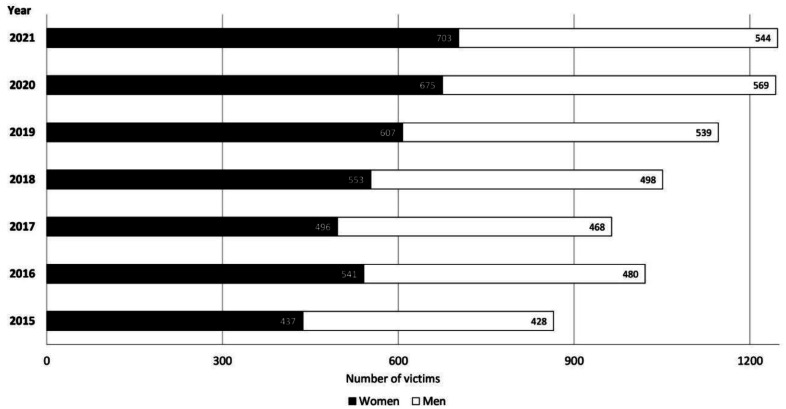
Number of male and female victims involved in hiking accidents in the Austrian Alps within a 7 year-period.

Overall, 7368 hiking accidents were registered in the Austrian Alps within a 7-year period (2015–2021). The accidents involved a total of 7552 victims. Figure 2 shows the annual numbers of victims distributed by sex, which increased by 44% from 865 victims in 2015 to 1247 victims in 2021.

Overall, 4012 women (53.1%) and 3526 men (46.7%) were involved in the reported hiking accidents. Sex was either unknown or not specified in 14 victims (0.2%; not shown in the graphs). Age was reported in 7520 male and female victims (99.6%), with an average of 52.6 (±18.5) years. Age was not specified in 32 victims (0.4%). Descriptive sex and age distributions combined with the outcome of the accident (fatal/non-fatal/unknown) are shown in Table 1.

**Table 1.** Total number of victims characterized by age, sex, fatal, non-fatal or unknown accident outcome.

**Age in years**		**≤15**	**16–34**	**35–54**	**55–74**	**≥75**	**Total**
**All victims**	n (%)						
Women		132 (3.3)	563 (14.1)	1255 (31.4)*	1689 (42.3)*	352 (8.8)	3991 (53.2)
Men		170 (4.8)	570 (16.2)	928 (26.4)	1322 (37.6)	527 (15.0)*	3517 (46.8)
Total		302 (4.0)	1133 (15.1)	2183 (29.1)	3011 (40.1)	879 (11.7)	7508 (100.0)
**Fatalities**	n (%)						
Women		3 (2.9)	16 (15.7)	30 (29.4)	42 (41.2)	11 (10.8)	102 (19.2)
Men		3 (0.7)	26 (6.1)	86 (20.1)	230 (53.9)	82 (19.2)*	427 (80.8)*
Total		6 (1.1)	42 (7.9)	116 (21.9)	**272 (51.5)†**	93 (17.6)	529 (7.1)
**Non-fatalities**	n (%)						
Women		121 (3.3)	514 (13.9)	1164 (31.5)	1572 (42.5)	325 (8.8)*	3696 (55.9)*
Men		161 (5.5)	520 (17.8)	797 (27.3)	1023 (35.1)	414 (14.3)	2915 (44.1)
Total		282 (4.2)	**1034 (15.7)†**	1961 (29.7)	2595 (39.2)	739 (11.2)	6611 (88.0)
**Unknown**	n (%)						
Women		8 (4.1)	33 (17.1)	61 (31.6)	75 (38.9)	16 (8.3)	193 (52.5)
Men		6 (3.4)	24 (13.7)	45 (25.7)	69 (39.4)	31 (17.8)	175 (47.5)
Total		14 (3.8)	57 (15.5)	106 (28.8)	144 (39.2)	47 (12.7)	368 (4.9)

Notes: significant differences (p ≤ 0.001) between sexes* and age categories† in association with fatal or non-fatal outcomes; total numbers do not include cases in which sex or age was unknown.

When examining age categories in association with the sex of all victims, a chi-square test of independence revealed significant sex differences regarding the age categories 35 to 54, 55 to 74, and ≥75 years (χ2 = 103.9, 4 df, p ≤ 0.001, V = 0.1). Post-hoc analyses showed that men aged ≥75 years were 1.5 times more likely to have an accident than women, whereas women were 1.4 times more likely to have an accident when aged between 35–54 and 1.3 times more likely when aged between 55–74 years (χ2 = 103.9, 4 df, p ≤ 0.001).

When examining fatal and non-fatal outcomes in association to age and sex, significant differences were observed in both categories (age: χ2 = 77.44, 8 df, V = 0.07 and sex: χ2 = 265.6, 2 df, V = 0.2). According to the post-hoc analyses, victims between the age of 16 and 34 years were 43.1 times more likely to have non-fatal accidents and victims between the age of 55 and 74 years were 9.5 times more likely to have fatal accidents (χ2 = 77.44, 8 df, p ≤ 0.001). No significant differences were found within the age categories <15 years, 35 to 54, and ≥75 years (χ2 = 77.44, 8 df, p > 0.05). Post-hoc analyses according to sex showed that men were 4.2 times more likely to have fatal accidents, and women were 1.3 times more likely to have non-fatal accidents (χ2 = 265.6, 2 df, p ≤ 0.001).

### 3.2. Injury degrees in non-fatal accident outcomes

Within the category of non-fatal accidents, three categories of injury degrees were formed: slightly injured, severely injured, and an unknown degree of injury. The descriptive age and sex distributions of injury degrees are summarized in Table 2.

Significant sex differences were observed within injury degrees (χ2 = 333.8, 3 df, V = 0.21). Women were 1.0 times more likely to be slightly injured (50.1 %) and 1.6 times more severely injured (62.1 %) than men (χ2 = 333.8, 3 df, p ≤ 0.001). No significant sex differences were found in those victims with injuries to an unknown degree (χ2 = 333.8, 3 df, p > 0.05). When examining age categories in association to injury degrees, significant differences were found (χ2 = 143.22, 8 df, V = 0.10). Victims in the age category between 35 and 54 years, as well as between 55 and 74 years, were 1.0 times and 1.3 times more likely to be severely injured, respectively (χ2 = 143.22, 8 df, p ≤ 0.001). In the other categories, no statistically significant differences were found (χ2 = 143.22, 8 df, p > 0.05).

**Table 2.** Sex and age distribution of injury degrees in non-fatal accident outcomes.

**Age in years**		**≤15**	**16–34**	**35–54**	**55–74**	**≥75**	**Total**
**Non-fatalities**							
**Slightly injured**	n (%)						
Women		56 (4.6)	223 (18.5)	377 (31.2)	426 (35.3)	125 (10.4)	1209 (50.1)*
Men		79 (6.5)	234 (19.4)	309 (25.6)	386 (31.9)	201 (16.6)	1207 (49.0)
Total		135 (5.6)	457 (19.0)	686 (28.3)	812 (33.6)	326 (13.5)	2416 (100.0)
**Severely injured**	n (%)						
Women		25 (1.7)	131 (9.1)	450 (31.3)	713 (49.6)	118 (8.3)	1437 (62.1)*
Men		36 (4.1)	133 (15.2)	268 (30.6)	353 (40.3)	87 (9.8)	877 (37.9)
Total		61 (2.6)	264 (11.4)	**718 (31.1)†**	**1066 (46.1)†**	205 (8.8)	2314 (100.0)
**Unknown**	n (%)						
Women		40 (3.8)	160 (15.2)	337 (32.0)	433 (41.2)	82 (7.8)	1052 (55.9)
Men		46 (5.5)	153 (18.5)	220 (26.5)	284 (34.3)	126 (15.2)	829 (44.1)
Total		86 (4.4)	313(16.5)	557 (29.5)	717 (38.2)	208 (11.4)	1881 (100.0)

Notes: significant differences (p ≤ 0.001) between sexes* and age categories† in association to injury degrees; total numbers do not include cases in which sex or age was unknown.

### 3.3. Pathophysiological characteristics

The pathophysiological characteristics of victims included fractures, fatigue, cardiovascular diseases, contusions/strains/ruptures, multiple types of injuries (injuries of at least two regions of the body), subluxations/sprains/dislocations, wounds/blood loss, and unknown/unspecified injuries. The absolute and relative frequencies of the total sample, as well as sex specific distributions of pathophysiological characteristics of victims, are summarized in Table 3.

**Table 3.** Distribution of pathophysiological characteristics of all victims.

**N (%)**	**Women**		**Men**		**Total**	
**Pathophysiological characteristics**						
Contusions/strains/ruptures	1251	(16.6)	924	(12.1)	2175	(28.9)
Fractures	**1253**	(16.6)*	576	(7.6)	1829	(24.3)
Wounds/blood loss	440	(5.8)	**592**	(7.9)*	1032	(13.7)
Unknown/unspecified injuries	454	(6.0)	444	(5.9)	898	(11.9)
Multiple types of injuries	268	(3.8)	**405**	(5.4)*	673	(8.9)
Cardiovascular diseases	84	(1.1)	**307**	(4.1)*	391	(5.2)
Subluxation/sprains/dislocations	183	(2.4)	175	(2.3)	358	(4.7)
Fatigue	79	(1.0)	103	(1.4)	182	(2.4)
Total	4012	(53.3)	3526	(46.7)	7538	(100.0)

Notes: *significant differences (p ≤ 0.001) within sex categories in association to pathophysiological characteristics of victims; fatigue: being unable to continue hiking due to overall physical and mental exhaustion; cardiovascular diseases: coronary heart disease, cerebrovascular disease, peripheral arterial disease, rheumatic heart disease, congenital heart disease, deep vein thrombosis and pulmonary embolism [8]; multiple types of injuries: injuries of at least two regions of the body; unknown/unspecified injuries: no information on the injury provided by the Austrian Alpine Police; total numbers do not include cases with unknown sex.

Significant sex differences were found within these victims' characteristics (χ2 = 473.8, 9 df, V = 0.25). According to the post-hoc comparisons, women were 2.2 times more likely to suffer from fractures, whereas men were 3.7 times more likely to suffer from cardiovascular events, 1.5 times more likely to suffer from multiple types of injuries, and 1.3 times more likely to suffer from wounds/blood loss (χ2 = 473.8, 9 df, p ≤ 0.001).

### 3.4. Injury locations

**Figure 3.**
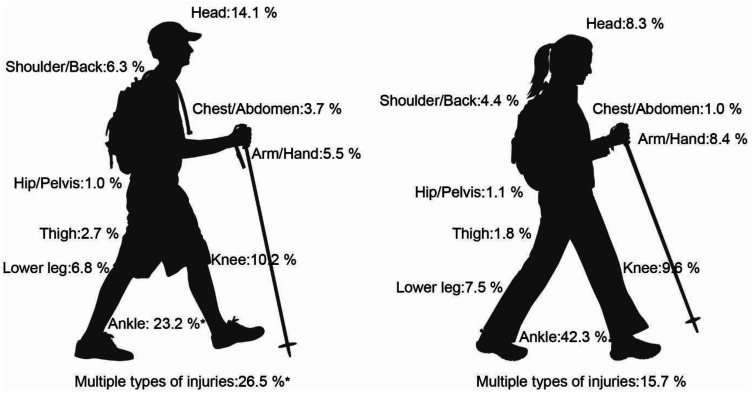
Percentage of injury locations for non-fatal hiking accidents in female and male mountain hikers. *p ≤ 0.05 compared to female participants.

The injury locations included “multiple body parts”, mostly affected by “multiple types of injuries”, as well as the upper (head, shoulder/back, chest/abdomen, arm/hand) and lower extremities (hip/pelvis, thigh, lower leg, knee, and ankle). The total numbers and sex specific distributions of the injury locations are shown in Figure 3.

Significant sex differences were present in these categories (χ2 = 428.9, 9 df, V = 0.25). According to post-hoc comparisons, men were 3.1 times more likely to injure the abdomen/chest, 1.4 times more likely to injure the head, and 1.1 times more likely to injure multiple body parts, whereas women were 2.2 times more likely to injure the ankle (χ2 = 428.9, 9 df, p ≤ 0.001).

### 3.5. Causes of injuries

Injuries were caused by either stumbling/tripping/falling, falling from heights, cardiovascular events, or other reported factors such as lack/failure of equipment, getting lost, sudden weather change/lightening, undefined diseases, fatigue, falling rocks, more than one of the mentioned causes, other causes, and unknown causes. The absolute and relative frequencies of the total sample, as well as sex specific distributions of the injury causes, are shown in Table 4.

**Table 4.** Sex specific distribution of all reported injury causes.

**N (%)**	**Women**		**Men**		**Total**
**Causes of injuries**			
Stumbling/tripping/falling	**3249**	(43.1)*	2344	(31.1)	5593	(74.2)
Cardiovascular events	83	(1.1)	**347**	(4.6)*	430	(5.7)
Other cause	150	(2.0)	159	(2.1)	309	(4.1)
Unknown cause	125	(1.7)	123	(1.6)	248	(3.3)
Fatigue	107	(1.4)	118	(1.6)	225	(3.0)
Falling from heights	71	(0.9)	**144**	(2.0)*	215	(2.9)
Falling rocks	84	(1.1)	83	(1.1)	167	(2.2)
Getting lost	53	(0.7)	78	(1.0)	131	(1.7)
More than one of the mentioned causes	45	(0.6)	64	(0.8)	109	(1.4)
Undefined diseases	25	(0.3)	45	(0.6)	70	(0.9)
Lack/failure of equipment	11	(0.2)	8	(0.1)	19	(0.3)
Sudden weather change/lightening	9	(0.1)	13	(0.2)	22	(0.3)
**Total**	4012	(53.2)	3526	(46.8)	7538	(100.0)

Notes: *significant differences (p ≤ 0.001) within sex categories in association to injury causes; cardio-vascular events: heart attacks or strokes; fatigue: being unable to continue hiking due to overall physical and mental exhaustion; undefined diseases, other cause, unknown cause, more than one of the mentioned causes: categorization of the Austrian Alpine Police without further specification; total numbers do not include cases with unknown sex or age.

The data showed that women were 1.4 times more likely to have an accident due to stumbling/tripping/falling than men (χ2 = 319.1, 11 df, V = 0.21). On the other hand, men were 2.0 times more likely to fall from heights and to have an accident due to cardiovascular events (χ2 = 319.1, 11 df, p ≤ 0.001).

### 3.6. Accident conditions and path characteristics

Accidents occurred either during ascent, descent, or during other conditions (e.g., walking on flat terrain). Significant sex differences within accident conditions showed that women were 1.2 times more likely to have an accident during the descent and men were 1.1 times more likely during the ascent (χ2 = 33.3, 3 df, V = 0.06, p ≤ 0.001). Accidents occured on marked hiking trails, on small paths, in a pathless terrain, on driveways or forest roads, and in other terrain or unknown conditions. Significant sex differences within these conditions revealed that men were 1.7 times more likely to have accidents in pathless terrains (χ2 = 87.1, 5 df, V = 0.10, p ≤ 0.001). The absolute and relative frequencies of the total sample, as well as sex specific distributions, are shown in Table 5.

**Table 5.** Accident conditions and path characteristics.

**N (%)**	**Women**		**Men**		**Total**	
**Hike characteristics**			
Accident during descent	**2769**	(69.0)*	2231	(63.3)	5000	(66.3)
Accident during ascent	770	(19.2)	**851**	(24.1)*	1621	(21.5)
Others (e.g. walking on flat terrain)	218	(5.4)	186	(5.3)	404	(5.4)
Unknown	255	(6.4)	258	(7.3)	513	(6.8)
**Total**	4012	(53.2)	3526	(46.8)	7538	(100.0)
Marked hiking trails	1570	(20.8)	1220	(16.2)	2790	(37.0)
Small paths	553	(7.3)	556	(7.3)	1109	(14.7)
Pathless terrain	211	(2.8)	**367**	(4.9)*	578	(7.7)
Drive way, forest roads	178	(2.4)	132	(1.8)	310	(4.1)
Other terrain	27	(0.4)	34	(0.5)	61	(0.8)
Unknown conditions	1473	(19.5)	1217	(16.1)	2690	(35.7)
**Total**	4012	(53.2)	3526	(46.8)	7538	(100.0)

Notes: *significant differences (p ≤ 0.001) within sex categories in association to accident conditions (descent, ascent, others or unknown) and path characteristics; total numbers do not include cases with unknown sex or age.

### 3.7. Alcohol intake of victims

Data on alcohol intake was only available for the years 2015 to 2019. Therefore, only 4616 victims (61.1 % of the total sample) could be accounted for these analyses. Among the subsample, 64 victims (1.4 %) consumed alcohol on the day of the accident (20.3 % women and 79.7 % men). Significant sex differences showed that men were 3.9 times more likely to consume alcohol on the day of the accident than women (χ2 = 26.7, 1 df, Φ = 0.08; p ≤ 0.001). Further analyses showed that there was no significant association between a fatal outcome of the accident and alcohol intake (χ2= 0.01, 1 df, Φ = 0.00; p > 0.976).

## 4. Discussion

This study provides a sex specific update of hiking accidents between the years 2015 and 2021 in the Austrian Alps. The results confirmed that the number of non-fatal hiking accidents in both men and women had been rising continuously, especially during the summer months of 2020 and 2021, against the background of lockdown and entry restrictions due to the COVID-19 pandemic. This constant and substantial increase in hiking victim numbers for the period from 2015 (437 women, 428 men; 865 in total) to 2021 (703 women, 544 men; 1247 in total) reported in the present study (see Figure 1) supports the suggestion of increasing accident numbers among hikers as stated by Faulhaber et al. (2017) [7]. The authors did not report sex-specific differences in hiking accidents, though they presented data on hiking accidents for the period from 2006 to 2014 and found a constant increase of total hiking accident numbers from 511 persons in 2006 to 733 persons in 2014.

On the other hand, it may be possible that restrictions in organized sport activities during the pandemic led to an increase in non-organized outdoor sports and physical activity for the years 2020 and 2021 [11]. In general, the rising number of non-fatal hiking accidents could be ascribed to the rising popularity of mountain hiking, thereby considering the exponential increase in the past two decades [7],[8],[12]. Additionally, the continuously rising membership numbers of the German and Austrian Alpine Clubs in the past years could reflect the increasing popularity of mountain hiking in the Austrian Alps [13],[14].

Moreover, the present study confirmed data from alpine sports practiced in Austria during the summer season, i.e., mountain biking and hiking [7],[15], revealing men being more likely to suffer from fatal accidents than women. However, the number of fatal hiking accidents in the Austrian Alps has remained relatively stable (<10%) over the investigated time period and compared to data from scientific literature [5],[7]. A similar trend has been shown for fatal accident outcomes in other mountain sports such as mountain climbing [14]; concerning mountain biking, the results from literature seem to be inconsistent [15],[16]. In the present study, men suffered from multiple types of injuries and cardiovascular events more frequently, whereas women were more likely to sustain fractures mostly involving the lower body (e.g., the ankle or foot). Moreover, men tended to more likely injure the abdomen/chest or head. These sex specific characteristics concerning all hiking accidents between 2015 and 2021 provide a follow-up of the analyses by Faulhaber et al., (2017) [7] who descriptively displayed sex specific characteristics of injury locations in fall-related mountain hiking accidents between 2006 and 2014. Comparing the increasing number of victims between male and female hikers between 2015 and 2021, the larger increase of female victims becomes obvious (Figure 1). We assume this for different reasons. First, there is an increasing number of women participating in mountain sport disciplines in general, and in hiking in particular. This assumption is supported by the report of increasing numbers of female members in the European alpine clubs. For example, the proportion of female members in the German Alpine Club (“DAV”) increased from 40.4% in 2011 up to 43.6% in 2022 [17]. Second, several studies have shown that accident and injury patterns differ between males and females in multiple accident situations such as in skiing [18] or driving [19], and that the female sex can be a risk factor for falls and fractures in the elderly [20].

Falling/tripping or stumbling contributed to more than 70% of injury causes in the present study; however, women were more likely to have an accident due to these causes, whereas men were more likely to fall from heights. Furthermore, we can confirm the analysis of the previous 9-year period with the finding that men were more likely to have hiking accidents in pathless terrains, which has been previously shown to be fatal often [7]. This might be related to a possible enhanced risk-taking behavior in men, especially in male summer alpinists [16],[21]. However, this has mostly been investigated in mountain sports other than hiking (i.e., mountain running or climbing) [22]. Therefore, this argument could be confounded due to the generally higher number of male participants performing these activities [23].

In more than 60% of the victims, hiking accidents happened during the descent; however, they occurred more often in women than in men, as previously shown by Faulhaber et al. [7]. In order to explain the dynamic of the accidents, we can speculate that traumatic accidents during the descent might result in injured lower body parts mainly due to falling/tripping or stumbling more often, which was significantly more present in women. On the other hand, men tended to suffer more frequently from cardiovascular events, presumably mainly during the ascent, as shown in other summer alpine sports [15].

Furthermore, in an analysis of hiking accidents performed in the Swiss Alps between 2009 and 2018, Gasser (2019) and Faulhaber (2017) showed that elderly hikers tended to suffer from severe non-fatal accidents more frequently than younger hikers [7],[24]. The results of the present study showed that age categories did not statistically differ regarding the effect on the injury degree in non-fatal hiking accidents. However, we showed that women between the age of 35 and 74 were more likely to be severely injured compared to men. The comparison of the two studies could be confounded due to different categorization methods of the Swiss Scoring System of emergency medicine (NACA-Score) and the categorization into slight and severe injuries used by the Austrian Alpine Police [24].

Although data on alcohol intake could only be reported for 60% of all victims and only between the years 2015 and 2019, we found that men were more likely to consume alcohol than women. However, this only accounted for 2 % of those who reported alcohol intake on the day of the accident. We confirmed the results of the previous 9-year analysis that more than 90 % of victims didn't report alcohol intake on the day of the accident [7]. Due to the limited amount of data, this difference should not be over-emphasized. Moreover, the response bias due to socially desirable responding cannot be excluded.

In addition, we would like to address the limitation that the data of the Austrian Alpine Police is retrieved from a semi-standardized documentation system. Therefore, the types of categories within the domains of hiking circumstances, locations, types, injury degrees, and causes was the authors decision. However, we followed the pattern of previous studies, especially the one of Faulhaber et al., (2017) [7]. Furthermore, the data of alcohol intake is missing for the latest time period (2020 and 2021), which provides limited strength of the statement.

According to the previous 9-year analysis of hiking accidents in the Austrian Alps, Faulhaber et al. (2017) suggested that mountain hikers should be informed about the discovery that the descent is a risky part of hiking [7]. In addition to this recommendation, the novel information could also be used for scholarly purposes by Alpine Club organizations, so that individuals (e.g., mountain guides, hiking guides) are aware of these facts and could be sent out as multipliers in the field by educating participants of organized hiking trips about sex specific injury causes, applying injury prevention messages or offering route recommendations to other Alpine Club members. Moreover, specific exercises could be implemented at the beginning of organized hiking trips in order to practice descending. Additionally, these results could be shared with the Austrian Alpine Police and improvements of accident documentation as well as victim characteristics documentation could be discussed to further identify sex-specific risk factors. For future lines of research, a comparison of accident characteristics in different countries (e.g., Switzerland, Germany, Austria, Italy France) could be of significant value in order to provide similar risk management and prevention programs for the Alpine regions of these countries.

## 5. Conclusions

In summary, sex-specific analyses regarding circumstances, causes, and pathophysiological characteristics of hiking accidents have mainly been performed descriptively. Nevertheless, this paper contributes to a better understanding of sex-specific characteristics of mountain accidents during the past few years with the main findings that stumbling/tripping or falling might be the main causes for hiking accidents in the Austrian Alps, and that women could mostly be at risk for these injury causes. In order to improve injury prevention among mountain hikers, we suggest developing practical recommendations based on the following scheme: first, reduce fatalities (men might be at higher risk); second, reduce serious injuries (men might be at higher risk); and third, reduce minor injuries (women might be at higher risk). Alpine Club organizations could use this pattern connected with the sex-specific injury analyses to educate their multipliers.

## Use of AI tools declaration

The authors declare they have not used Artificial Intelligence (AI) tools in the creation of this article.
